# Medical Schools in the Provinces

**Published:** 1920-09-04

**Authors:** 


### MEDICAL SCHOOLS IN THE PROVINCES.

**Birmingham University (Faculty of Medicine).**—In order to obtain the degrees of M.B. and Ch.B. five examinations have to be passed. The first is in chemistry, physics, and elementary biology; the second in anatomy and physiology; the third in general pathology, bacteriology, and materia medica and pharmacy; the fourth in forensic medicine, toxicology, public health, therapeutics, and special pathology; and the fifth, or final, in medicine, surgery, midwifery, diseases of women, mental diseases, and ophthalmology. The University also grants degrees and a diploma in dental surgery, and a diploma (D.P.H.) and a degree in Public Health (B.Sc.). The General Hospital and the Queen's Hospital, containing between them over 500 beds, are amalgamated for the purpose of clinical instruction, which is carried on under the direction of the University Clinical Board. Medical students also have free access to clinical instruction at the Eye, Ear and Throat, Orthopædic and Spinal, Women's, and Children's Hospitals, which are associated with the University. The composition fee for the whole course of lectures and instruction at the University is £85, besides which there is a composition fee for attendance on the medical and surgical practice and the clinical lectures at both the General and Queen's Hospitals of £42, making a total of £127. Dean: Mr. William F. Haslam, F.R.C.S. The Winter Session opens on Monday, October 4, 1920.

**Bristol University.**—The University grants the conjoined degrees of M.B., Ch.B., the degrees of Doctor of Medicine (M.D.), Master of Surgery (Ch.M.), Bachelor of Dental Surgery (B.D.S.), and Master of Dental Surgery (M.D.S.)*; also diplomas in Public Health (D.P.H.), in Dental Surgery (L.D.S.),* and in Veterinary State Medicine (D.V.S.M.). For the conjoined degrees of M.B., Ch. B., the curriculum occupies five years and a half. The University lectures and laboratory courses are attended in the large and well-equipped new wing of the University. Hospital practice and clinical instruction are

* The fee for laboratory instruction in mechanical dentistry has been reduced to 40 guineas, which covers two years' instruction.

provided in the allied hospitals, which together afford upwards of six hundred beds and a very large external midwifery department. Three years at least must be spent in the University, and two of them subsequently to passing the second examination. Women are admitted to all degrees and diplomas and to the courses of study necessary for them. The winter session opens on October 1, 1919.

**Cardiff University College (School of Medicine).**—All classes are open to both men and women students. The courses of instruction given in the school of medicine at the University College, Cardiff, are recognised as qualifying for the examinations of the Universities, Royal Colleges, and other licensing bodies in Great Britain and Ireland, and they are specially adapted to meet the needs of those University students studying for Welsh and London degrees. Having spent two or three years in study at Cardiff, and having passed the corresponding examinations, a student may proceed to London or elsewhere and complete his qualifying course for a University degree or for a diploma. It will be possible, when the Welsh National School of Medicine is fully constituted, for students to pursue the complete curriculum at Cardiff. Students who are preparing for these examinations may compound for their courses by paying a fee of £63, while a composition fee of £41 10s. includes all the necessary courses for the first and second examinations for the diploma of the Conjoint Board. In all cases the composition fees may be paid by instalments. Hospital instruction is given at the King Edward VII.'s Hospital, Cardiff. The attention of students about to matriculate is drawn to the numerous entrance scholarships offered for competition at University College, Cardiff, in April next, most of which may be held by medical students. Full particulars of the examination for these may be obtained by application to the Registrar. In the Department of Public Health, established in 1899, instruction is given qualifying for the D.P.H. examinations. A course for the training of health visitors has been instituted. Further information may be obtained from the Dean of the Faculty of Medicine.

Clinical Instruction.—*King Edward VII.'s Hospital, Cardiff.*—Students can attend the practice of this hospital, which contains 450 beds. Founded 1837.

**Durham University.**—For students who intend to graduate at Durham University it is necessary that one of the five years of professional education should be spent in attendance at the College of Medicine, Newcastle-upon-Tyne. During the year so spent the student must attend lectures at the College and the medical and surgical practice and clinical lectures at the Royal Victoria Infirmary, which contains 600 beds. No residence at Durham is necessary, the Medical Faculty being in Newcastle-upon-Tyne. The remaining four years of the curriculum may be spent at Newcastle-upon-Tyne or at one or more of the recognised medical schools. There are four professional examinations for the M.B., B.S. degrees. Particulars as to fees, etc., may be obtained from Professor Howden, Registrar, College of Medicine, Newcastle-upon-Tyne.

**Leeds University (School of Medicine).**—This University grants the degrees of M.B. and Ch.B., M.D. and Ch.M., M.Ch.D. and B.Ch.D., and, in association with the Universities of Liverpool, Manchester, Sheffield, and Birmingham, holds a Matriculation examination. Clinical instruction is given in the General Infirmary, which contains 620 beds, and is amply provided with special departments. Students of the University also have access for instruction to the Leeds Public Dispensary, the City Fever Hospitals, the Hospital for Women and Children, the Leeds Maternity Hospital, and the West Riding Asylum at Wakefield. Diplomas in Public Health, Psychological Medicine, and Dental Surgery are also granted.

**Liverpool University (Faculty of Medicine).**—The University grants degrees in Medicine (M.B.; M.D.), in Surgery (Ch.B.; Ch.M.), and in Hygiene (M.H.). Candidates for degrees are required to pass the Matriculation examination or an equivalent. Students may study in the University for the degrees or for the examinations of other licensing bodies. The laboratories are modern and very well equipped, and the facilities for instruction are most complete. Courses of instruction in tropical diseases can be obtained at the Liverpool School of Tropical Medicine. Fellowships, scholarships, and prizes of over £1,000 are awarded annually, in addition to entrance scholarships. The Clinical School of the University now consists of four general hospitals—the Royal Infirmary, the David Lewis Northern Hospital, the Royal Southern Hospital, and the Stanley Hospital; and of five special hospitals—the Eye and Ear Infirmary, the Hospital for Women, the Infirmary for Children, St. Paul's Eye Hospital, and St. George's Hospital for Skin Diseases. These hospitals contain in all a total of about 1,134 beds. The organisation of these hospitals to form one teaching institution provides the medical student and the medical practitioner with a field for clinical education and study which is unrivalled in extent in the United Kingdom. The University also grants a licence and degrees in dental surgery, a diploma and degrees of Bachelor, Master, and Doctor in veterinary medicine and surgery, a diploma in public health, diploma in tropical medicine, and a diploma in medical radiology and electrology. The Dental Hospital was opened in January 1910, and its equipment for teaching purposes is complete.

**Manchester, Victoria University (Faculty of Medicine).**—The medical department of the University, which is open to both men and women students, is particularly well provided with facilities for instruction in all the courses for degrees and the professional qualification and for teaching the preliminary subjects—namely, anatomy, physiology, pathology, materia medica, biology, physics, and chemistry. Clinical instruction is provided at the Manchester Royal Infirmary, which contains 671 beds, and also has large out-patient and casualty departments. Associated with the Infirmary are a Convalescent Hospital at Cheadle, containing 136 beds, and the Royal Lunatic Hospital, accommodating 4[illegible] patients. Clinical instruction in gynæcology is also given at St. Mary's Hospital, and other hospitals.

**Sheffield University (Faculty of Medicine).**—The degrees of the University, M.B., Ch.B., M.D., Ch.M., and the diploma in public health are open to men and women alike. A candidate for the degrees M.B., Ch.B. must produce certificates that he will have attained the age of 21 years on the day of graduation, that he has pursued the courses of study required by the University Regulations during a period of not less than five years subsequently to the date of his matriculation or exemption from matriculation, three of such years at least having been passed in the University, one at least being subsequent to the passing of the first examination. The University is within easy reach of the various hospitals with which it is connected for clinical purposes. The composition fee for University lectures, practical class[illegible]

and hospital practice is £30 a year for each of the five years. The University of Sheffield offers many valuable scholarships and prizes to students in the faculty of medicine.

**Wales (University of)**—This University has the privilege of granting degrees in medicine and diplomas in public health. At the three constituent Colleges of Aberystwyth, Bangor, and Cardiff there are Professors of Chemistry, Botany, Zoology, and Physics, so that the students of the University can obtain proper instruction in the ancillary subjects. The foundation of a medical faculty has been laid at University College, Cardiff, where there is a recognised school of medicine (*see* p. 584).

